# Response to “Better late than never!—a long-awaited necessary turn in the prenatal management of early 2nd trimester LUTO”

**DOI:** 10.1007/s00467-026-07288-5

**Published:** 2026-04-07

**Authors:** Stefan Kohl, Jaap Mulder, Rik Westland

**Affiliations:** 1 Children’s and Adolescents’ Hospital, Faculty of Medicine and University Hospital Cologne, University of Cologne, Cologne, Germany; 2 Division of Nephrology, Department of Pediatrics, Sophia Children’s Hospital, Erasmus Medical Center, Rotterdam & Division of Nephrology, Department of Pediatrics, Willem-Alexander Children’s Hospital, Leiden, University Medical Center, Leiden, the Netherlands; 3 Department of Pediatric Nephrology, Amsterdam UMC-Emma Children’s Hospital, University of Amsterdam, Amsterdam, the Netherlands

Dear Editors,

We thank Dr. Kohl for his timely letter and for his longstanding commitment to advancing the prenatal management of fetuses with congenital lower urinary tract obstruction (cLUTO) [1]. Early implementation of vesico-amniotic shunting (VAS) in the first trimester would not have been possible without the pioneering efforts of a few dedicated fetal interventionists, including him.

However, pioneering spirit alone is not sufficient for the responsible clinical implementation of a new therapeutic paradigm. Early VAS in the late first or early second trimester represents more than a technical refinement—it entails intervention at a developmental stage with incompletely understood long-term consequences [2].

Initial retrospective reports of early VAS provided insufficient prenatal and postnatal follow-up data for transparent evaluation of outcomes. This lack of prospective data understandably contributed to divergent views regarding early VAS, particularly among clinicians shaped by earlier experience with fetal shunting before the PLUTO trial.

To address this, two of the authors of the review initiated the prospective IUS1st study, to generate systematically collected outcome data after early VAS for severe cLUTO [3]. These data suggest potential benefits, including encouraging early kidney function in many survivors and mostly self-limiting complications, while also revealing substantial urological morbidity and complex long-term outcomes.

Prospective studies and registry-based data are essential for clinical decision-making and responsible expectation management during prenatal counseling. Ethical responsibility in fetal therapy requires careful multidisciplinary counseling and transparent communication of benefits, risks, and uncertainties to enable informed shared decision-making.
